# Supplementary material for: Influence of intrauterine factors on birth weight and on child linear growth in rural Ethiopia: A prospective cohort study
Source: PLoS One. 2022 Aug 5;17(8):e0272620. doi: 10.1371/journal.pone.0272620 (PMC9355213; doi:10.1371/journal.pone.0272620)
Supplement: Attachment — Submitted filename: Response to Reviewers.docx [file pone.0272620.s001.docx]

**Author's response to reviews**

**Title:** Influence of intrauterine factors on birth weight and on child linear growth in rural Ethiopia:  a prospective cohort study

**Authors:**

Meselech Roro Mrs. ([meselua@yahoo.com](mailto:meselua@yahoo.com))

Wakgari Deressa Prof ([deressaw@gmail.com](mailto:deressaw@gmail.com))

Bernt Lindtjørn Prof ([bernt.lindtjorn@uib.no](mailto:bernt.lindtjorn@uib.no))

**Version: 2**	**Date: 06** July 2022

**Author's response to reviews:**

Dear Editor-In-Chief,

Thank you very much for sending us yours’ and the reviewers’ comments on our manuscript [Roro et al; MS: PONE-D-21-30564

Influence of intrauterine factors on birth weight and on child linear growth in rural Ethiopia:  a prospective cohort study].

First, we would like to express our appreciation for reviewers for their extensive evaluation of the manuscript and their critical comments. The comments were very helpful for making the paper clearer and more suitable for readers. We have revised the manuscript based on the comments accordingly and hope that we have addressed all of the comments to your satisfaction. We have also ensured that our revised manuscript conforms to the Journal style following the Journal formatting guidelines.

Attached please find a point- by -point response as well as the revised manuscript with highlighted track changes 'Revised Manuscript with Track Changes' and without track changes 'Manuscript'.

We would also like to inform you that the texts in track changes are the modification made in the manuscript and the line number written in the point- by – point response are the numbers in manuscript without track change, the ‘Manuscript’.

If you have any further queries, please do not hesitate to contact us at the contact address given below.

Best regards

Meselech Assegid Roro

Email: [meselua@yahoo.com](mailto:meselua@yahoo.com)

Corresponding Author

**Response to the Academic Editor:**

All the responses are in boldface**.**

1. Please ensure that your manuscript meets PLOS ONE's style requirements, including those for file naming. The PLOS ONE style templates can be found at
   https://journals.plos.org/plosone/s/file?id=wjVg/PLOSOne_formatting_sample_main_body.pdf and 
   <https://journals.plos.org/plosone/s/file?id=ba62/PLOSOne_formatting_sample_title_authors_affiliations.pdf>

**Response: The revised manuscript has followed the direction.**

2. In your Data Availability statement, you have not specified where the minimal data set underlying the results described in your manuscript can be found. PLOS defines a study's minimal data set as the underlying data used to reach the conclusions drawn in the manuscript and any additional data required to replicate the reported study findings in their entirety. All PLOS journals require that the minimal data set be made fully available. For more information about our data policy, please see http://journals.plos.org/plosone/s/data-availability.

Upon re-submitting your revised manuscript, please upload your study’s minimal underlying data set as either Supporting Information files or to a stable, public repository and include the relevant URLs, DOIs, or accession numbers within your revised cover letter. For a list of acceptable repositories, please see http://journals.plos.org/plosone/s/data-availability#loc-recommended-repositories. Any potentially identifying patient information must be fully anonymized.

Important: If there are ethical or legal restrictions to sharing your data publicly, please explain these restrictions in detail. Please see our guidelines for more information on what we consider unacceptable restrictions to publicly sharing data: http://journals.plos.org/plosone/s/data-availability#loc-unacceptable-data-access-restrictions. Note that it is not acceptable for the authors to be the sole named individuals responsible for ensuring data access.

We will update your Data Availability statement to reflect the information you provide in your cover letter.

**Response: The data was uploaded and the website link was made available on the Open Science Framework (OSF). Here is the link to the data:** [osf.io/t9n8q](http://osf.io/t9n8q)

3. We note that you have included the phrase “data not shown” in your manuscript. Unfortunately, this does not meet our data sharing requirements. PLOS does not permit references to inaccessible data. We require that authors provide all relevant data within the paper, Supporting Information files, or in an acceptable, public repository. Please add a citation to support this phrase or upload the data that corresponds with these findings to a stable repository (such as Figshare or Dryad) and provide and URLs, DOIs, or accession numbers that may be used to access these data. Or, if the data are not a core part of the research being presented in your study, we ask that you remove the phrase that refers to these data.

**Response: We have removed the phrase that says “data not shown” in both “Manuscript” and “Revised manuscript with track change”.**

4. Please upload a new copy of Figure 1 as the detail is not clear. Please follow the link for more information: https://blogs.plos.org/plos/2019/06/looking-good-tips-for-creating-your-plos-figures-graphics/" <https://blogs.plos.org/plos/2019/06/looking-good-tips-for-creating-your-plos-figures-graphics/>

**Response: We have prepared Figure 1 using PACE as per recommendation by PLOS and uploaded the new copy.**

5. Please upload a copy of Figure 3, to which you refer in your text on page 11. If the figure is no longer to be included as part of the submission please remove all reference to it within the text.

**Response: Reference that refers to Figure 3 is now removed from the text.**

6. Please review your reference list to ensure that it is complete and correct. If you have cited papers that have been retracted, please include the rationale for doing so in the manuscript text, or remove these references and replace them with relevant current references. Any changes to the reference list should be mentioned in the rebuttal letter that accompanies your revised manuscript. If you need to cite a retracted article, indicate the article’s retracted status in the References list and also include a citation and full reference for the retraction notice.

[Note: HTML markup is below. Please do not edit.]

**Response: We have checked the references and ensured that they are complete and correct.**

**B. Reviewer #1 Comments and Responses**

|  | Comments | Revision and response |
| --- | --- | --- |
| 1 | The title of the manuscript clearly expresses what the study is about and highlights the importance of the study. Although the abstract presents the aim, methods, important findings, and conclusion, it appears too long as the word count exceeds the specified word limit. As such, summarizing the sub-sections will improve the abstract. | Thank you for the comment. The abstract section is now more summarized. |
| 2 | Data analysis is appropriate. However, the measure of central tendency ('mean') used to summarize the following variables gravidity, family size, and parity, is inappropriate as they are not continuous variables. Suggestion: Mode. | Thank you for the comment. The comment is well accepted and the measures of central tendency were corrected. The study participants had median of 6 with (3 IQR) for family size, , 4 (4 IQR for gravida and 2 (4 IQR) for parity.  Indicated in line 352. |
| 3 | The methods are appropriate for the research questions but the final sample size after exclusion at different stages is less than the calculated minimum sample size. See lines 259-268 and 275. 'increased' non-response rate during sample size calculation would have been helpful in dealing with the above challenge. | Thank you. Thank you, this is a very important comment. Even though we enrolled 727 pregnant women, which is the calculated size in this prospective cohort study, at the end of the 29 months follow up period there were 554 children with complete data who we followed starting from foetal period through delivery and to 11-24 months postnatal period. The lost to follow up rate was more than what we anticipated in the sample calculation. To check if the sample size was still adequate enough, we calculated the power for the non-significant variables of interest such as wealth status (where we based our sample size calculation) and other confounders. However, we calculated the power for some non-significant results and found it to be low. This means that we could have missed some significant findings due to low sample size.  The comment is addressed in the limitation section as: “The final sample size was less than the calculated minimum sample size at the beginning of the study because of exclusion at different stages of the study that may have resulted in non-significance associations. This may indicate that we may have missed some risk factors for the child being stunted. Line 527-532 |
| 4 | Kindly limit the use of first-person pronouns in the report ( e.g see Lines 91,97 and 106 in the manuscript). Rephrasing such sentences may improve the manuscript. | Thank you. The comment is accommodated, and we have rephrased the sentences. |

**C. Reviewer #2 Comments and Responses**

|  | **Comments** | **Revision and response** |
| --- | --- | --- |
|  | General comments |  |
| 1 | The paper is well designed but the manuscript sections have to be consistent and some edit need to attract attention from the authors. Method section should need more details to define variables and to explain the statistically data analysis | Thank you for critical evaluation of the section. We have accepted the comments and added details. |
| 2 | The authors should pay more attention to the Results section and subsequently Discussion section. | Thank you for the comment. We have accommodated the comments in the result and discussion sections. |
| 3 | Format and consistency challenges need few restructuration. However, the author should pay more attention to be consistent with definitions and spelling of key variables. | Thank you for the comment. We have addressed the definitions and spelling of variables. |
| 4 | The effect size estimates, their values and the significance level are missing in the text and tables. | Thank you for the comment and we appreciate the critical review. We have included the effect size estimates and the significance level (text & tables) in line 383-388, 410-419 and 437 -439. |
|  | Specific comments |  |
|  | study design section |  |
| 1 | Lines 106-108: avoid repetitions in the sentence that doesn’t add value. “…that influence growth…. That affect linear growth…” | Thank you. The one “…. that affect linear growth of children…” was deleted |
| 2 | Lines 110 to 112: this sentence can be restructured in one sentence. “…foetal and child factors including foetal factors (HC, BP…) and birth characteristics (age, sex) …” | Thank you. It was restructured as “The foetal and child factors include foetal factors (HC, BPD, AC and FL) and birth characteristics (gestational age at birth and child sex). In line 135- 141. |
| 3 | Anthropometric measurements: Authors should provide how the quality control and precision have been done for maternal and child anthropometric measurements. | Trained nurses recorded the anthropometric measurements. They measured height of each pregnant woman at enrolment using a standard wooden board. Before starting the study, they were given two days of training on anthropometry measurement techniques. We checked inter-and intra-technical measurement errors, and gave repeated training until the measurements reached the recommended cut-off points.  … The data collectors for the child anthropometry in this study were the nurses who were trained and their measurement techniques standardized in a study that was being carried out at the same time in under five children in the same area. The intra and inter technical errors of measurements (TEM) were within the acceptable cut-off points. They were given additional one-day refreshment training before initiation of data collection. Before taking weight measurements, the scales were checked and calibrated. Birth weight was measured within 72 hours of delivery to minimize variations related with physiological changes that occur after 72 hours. Line 210-236.  However, after completing this study, we still found some measurement errors and had to delete these cases because they represented "extreme outliers". This is the reason for our limited sample size. |
| 4 | Nothing is said about how the wealth status has been computed. Which assets have been included for principal component analyses? | Thank you. We have accommodated the comment as “Wealth status was computed using fourteen household assets. The detail has been presented elsewhere. ( Ref. Roro et.al” Line 186 -188 and 256-257. |
|  | Statistical analysis |  |
| 1 | What was the added value to use two software (SPSS and Stata) for the same results, the same study? Using two software, how the authors addressed the conflicting statistical results between them? | Thank you for the comment. We used SPSS for data entry and cleaning. While we used STATA for data analysis.  We have rewritten the sentence as “Trained clerks entered the data from the paper-based standardized questionnaires and forms using SPSS version 24 (SPSS Inc., Chicago, IL, USA). Data were cleaned using SPSS statistical software. We used Stata software version 15 (Stata Corp. College Station, TX, USA) for analysis.”  It was state by mistake. We used SPSS only for data entry and Stata software version 15 (Stata Corp. College Station, TX, USA)was used for analysis. Line 250-251. |
| 2 | Lines 212-214: the author needs to add the third undernutrition status which is underweight related to weight-for-height z-score index. | Thank you again for the comment. Underweight is included in the sentence in line 265. |
| 3 | Lines 233-242: do gender and sex variables have the same meaning in the text? | Thank you. “Gender” is now replaced by “sex” in all sections of the document. |
|  | Participants section: |  |
| 1 | Line 126 should be edited: “…have been …” | Thank you. It is now edited |
| 2 | The author has to state based on the evidence with references and not from news in the first line of the paragraph | Thank you. We have added reference in the subsequent paragraph. Line 166 |
| 3 | References must be inserted for the follows section of the paragraph: “….were postponed.” | Thank you. But this comment is not relevant to our manuscript. |
| 4 | Paragraph 3: the author must avoid repetitions in the text such the aim in lines 5 and 10. And please, restructure the sentence: “…to answer these questions, this study aims to understand the experiences…” | Thank you. But this comment is not relevant to our manuscript. |
| 5 | The author has to remove bold and bullets from the specific objectives section. To understand the level of the stress, I doubt that the author can use a quantitative analysis as mentioned in the following method section. The author can use another verb. | Thank you. But this comment is not relevant to our manuscript. |
|  | Methods |  |
| 1 | Setting section (general setting and specific setting) has to be merged, shortened, and more accurate. Some other information is not relevant to the study aims and the study setting section such as the human index. The meaning of “lakh” seems confused and has to be reworded. The author has to delete the section that does not add any value:” …and is still in place with relaxations in the state.” | Thank you. But this comment is not relevant to our manuscript. |
| 1 | Study population: Eligibility criteria according to age must be inserted in this section. The interview was either semi-structured (method section) or structured (abstract). Please, clarify the interview type? | Thank you. But this comment is not relevant to our manuscript. |
|  | Variables, data source and data collection: |  |
| 1 | Definitions and the meaning and categories of all variables (exposures and outcomes) are missing. It is clear shown the unit for variables instead of the clear definitions of them. Authors should define variables clearly. For example, writing like “family size (number)” is confusing and not sufficient either defining family size or precising the unit of family size data. | Thank you for the critical comment. We have added the definitions of household wealth status and family size under variables section as “Fetal biometry measured by ultrasound, sex of child , gestational age at birth (measured in weeks), maternal age, maternal education, maternal occupation, parity, maternal height, household wealth status, family size and child age were the predictor variables in this study. A household wealth status was constructed by using principal component analysis as described previously (14). The number of household family members were used for family size.”  The detailed description of the outcome variables were presented under statistical analysis. Line 184-188 |
| 2 | In the method section, authors should clarify how data have been collected, aggregated and analysed to express Household wealth through principal component analysis method. | Thank you. We have now clarified the Household wealth as follows. “A household wealth status was constructed by using principal component analysis as described previously.” We have added a reference from previous publication. Line 186-188 |
| 3 | The collectors of data are not mentioned in the study, and which measurements they used to collect data and each variables. Authors should provide how they develop the quality control for measurement for their accuracy and reproducibility during the study. | Thank you again. We have accommodated the comment. We have now included details regarding the data collectors, the measurements and quality control under the section “**Anthropometric measurements**” as follows:  Trained nurses recorded the anthropometric measurements. They measured height of each pregnant woman at enrolment using a standard wooden board. Trained nurses recorded the anthropometric measurements. They measured height of each pregnant woman at enrolment using a standard wooden board. Before starting the study, they were given two days of training on anthropometry measurement techniques. We checked inter-and intra-technical measurement errors, and gave repeated training until the measurements reached the recommended cut-off points. Within 72 hours of birth, the nurses measured birthweight, and recorded sex of the new-born. The weights and lengths of the children were recorded once at their age between 11–24 months. Length was measured in supine position (recumbent length) using a standard wooden board. Weight was measured to the nearest 100 gram using a digital scale. The data collectors for the child anthropometry in this study were the nurses who were trained and their measurement techniques standardized in a study that was being carried out at the same time in under five children in the same area. The intra and inter technical errors of measurements (TEM) were within the acceptable cut-off points. They were given additional one-day refreshment training before initiation of data collection. Line 220-236 |
| 4 | The author must avoid repetitions in the whole text. Repetition in line 4 and 12 in the first paragraph for variables and data sources through the data collection section. | Thank you. We have checked the whole section for redundancies and avoided repetitions. |
| 5 | The analysis must state the effects size estimates that have been used to express the results in the statistical analyses section (Multilevel, mixed effect regression). Did this statistical analysis fit with data and the study design (cohort study)? The confidence interval and level of significance are missing from the methods and results sections. | Thank you. We have described the effect size, fitting, confidence interval and level of significance in the statistical analysis section as follows “Fitting of the multilevel model was done after checking for the presence of clustering. Initially, we fitted a null, single-level (standard) regression model. Then we ran a null, multilevel model with the random individual effect. The calculated test statistics for model fit of outcome measures was strong (P*<*0.001). To estimate the unadjusted and adjusted coefficient (β) with a 95% confidence interval (CI), we fitted a multilevel model to account for clustering.” Line 279-284  We have also accommodated the comment in the result section. Line 383-388, 410-419 and 437 -439. |
|  | Results |  |
| 1 | Results are relevant but text section is not linked to the five tables. How has the author proceeded to the regression analyses? Nothing has been said about the binary and multivariable steps. How the author managed the confusion factors from the regression models? | Thank you. The text sections are linked with the respective tables.  We have accommodated the comment on regression analysis as detailed in the statistical analysis of the methods section as follows:  “We used a multilevel, linear regression analysis model. We first checked for distributions of the independent and outcome variables before fitting the model. Fitting of the multilevel model was done after checking for the presence of clustering. Initially, we fitted a null, single-level (standard) regression model. Then we ran a null, multilevel model with the random individual effect.  The multilevel, linear regression analysis model was fitted for each exposure variables separately with the outcome variable and the final model was fitted for all exposure variables together.” Line 274- 28 |
| 2 | Tables need to have the legend, confidence intervals, and symbols need to be explained. | Thank you for the comment. We have now included the legend and confidence intervals and explained symbols in all the tables. |
| 3 | Table 1 has some formats that need to be edited. What is the meaning of mean for household wealth? The summary of household wealth variable should consider poor and rich households instead of the mean in table 1. | Thank you. Table 1 is now edited and wealth status is presented as poor, middle and rich. Line 352 |
| 4 | Table 2 and 3: while we assume that twins and multiple pregnancy were excluded from the study, the authors should clarify disparate samples with more children compared to mothers. For instance, 1479 children in table 2 compared to 547 women in table 1. Otherwise, the sample “n” does confuse readers when we consider the study design which is a cohort study with loss to follow up participants from the enrolment to the last time in the pregnancy. We can’t compute the mean for the sum of number of the participants that we follow at different time-points. | Thank you for the comment.  We have tried to make table 2 clear, that 1479 refers to the number of biometric measurements, which were repeated measurements.  The number of women was 554. The data was complete in most of the variables. There were missing data in few variables. For example, maternal height was taken for 547 women. |
| 5 | Table 4: clearly, maternal education and child age at birth remain the key factor for birth weight (strong relationship). Child anthropometry is associated to birth weight too. This statement should be developed in the discussion. | Thank you. We have presented the association of child anthropometry, maternal education and child age with birth weight in the discussion section.  “Our study also found that fetal, pregnancy, and maternal factors were significantly associated with birthweight z-score.” In the first paragraph of the discussion section and  “Some of the factors associated with birth weight in our study were similar to findings of other studies. Studies conducted in Indonesia showed that maternal education, gestational age at birth, and sex were found to be determinants of birth weight. Similarly studies from India (44) and Nepal (45) found significant associations between gestational age at birth and birthweight. Our study also found that biparietal diameter at early pregnancy and head and abdominal circumference at late pregnancy were significantly associated with birthweight.” Line 499- 505 |
| 6 | Table 5: family size and child age are associated to LAZ and WAZ. The authors should provide the direction for this relationship. | Thank you. We have included the details in the result section. “Child LAZ increased with increasing family size.”  “Children from smaller family size had better WLZ.”  Line 418-419 |
| 7 | The first paragraph should insert the finding from tables 5 and 6 as stated in this review. Was there collinearity between maternal education and child age at birth or birth weight? | Thank you. We have added it to the first paragraph of the discussion section “Our study also found that fetal, pregnancy, and maternal factors were significantly associated with birthweight z-score.”  Line 463-465. |
|  | References |  |
| 1 | The use of capital letter in the titles (either for the first letter of the title or first letter for each word) should be consistent for the whole list of references. | Thank you for the comment. We have made the corrections throughout the references. |
| 2 | Check for the use of capital letters in titles, abbreviation for the journal, the consistency of pages. Some references don’t have number of titles. | Thank you for the comment. We have made the corrections on the letters, abbreviations and titles. |
| 3 | Reference 19 must be complete. | Thank you. We have made the correction. |
